# The efficacy of autologous matrix-induced chondrogenesis (AMIC) for osteochondral lesions of the talus in the mid-long term: a systematic review and meta-analysis

**DOI:** 10.1186/s13018-024-04864-z

**Published:** 2024-06-24

**Authors:** Ning Jiang, Haomin Li, Jia Wang, Lin Shen, Xiantie Zeng

**Affiliations:** 1 Foot and Ankle Surgery, Tianjin Hospital, Tianjin, 300211 China; 2 Graduate School, Tianjin University of Traditional Chinese Medicine, Tianjin, 301617 China

**Keywords:** Autologous matrix-induced chondrogenesis, AMIC, Osteochondral lesions of the talus, Meta-analysis

## Abstract

**Purpose:**

The objective of this study was to provide a comprehensive review of the existing literature regarding the treatment of osteochondral lesions of the talus (OLT) using autologous matrix-induced chondrogenesis (AMIC), while also discussing the mid-long term functional outcomes, complications, and surgical failure rate.

**Methods:**

We searched Embase, PubMed, and Web of Science for studies on OLT treated with AMIC with an average follow-up of at least 2 years. Publication information, patient data, functional scores, surgical failure rate, and complications were extracted.

**Results:**

A total of 15 studies were screened and included, with 12 case series selected for meta-analysis and 3 non-randomized controlled studies chosen for descriptive analysis. The improvements in the Visual Analog Scale (VAS), the American Orthopaedic Foot & Ankle Society (AOFAS) ankle-hindfoot, and Tegner scores at the last follow-up were (SMD = − 2.825, 95% CI − 3.343 to  − 2.306, *P* < 0.001), (SMD = 2.73, 95% CI 1.60 to 3.86, *P* < 0.001), (SMD = 0.85, 95% CI 0.5 to 1.2, *P* < 0.001) respectively compared to preoperative values. The surgery failure rate was 11% (95% CI 8–15%), with a total of 12 patients experiencing complications.

**Conclusion:**

The use of AMIC demonstrates a positive impact on pain management, functional improvement, and mobility enhancement in patients with OLT. It is worth noting that the choice of stent for AMIC, patient age, and OLT size can influence the ultimate clinical outcomes. This study provides evidences supporting the safety and efficacy of AMIC as a viable treatment option in real-world medical practice.

## Introduction

Osteochondral lesion of the talus (OLT) primarily affects the articular surface and subchondral bone of the talus. As there is no soft tissue attachment to the talus surface, approximately 70% of primary OLT cases are associated with ankle injuries, such as sprains and fractures [1, 2]. Additionally, vascular diseases, infections, hormone disorders, and ossification disorders can also contribute to the development of OLT [3]. Due to inadequate blood supply to the talus osteochondral, the limited regenerative capacity following injury often renders conservative treatment ineffective, ultimately necessitating surgical intervention.

Microfracture (MF) is the most commonly employed surgical intervention for OLT. This procedure utilizes a special MF tool to create small perforations in the subchondral bone, facilitating the egress of bone marrow and blood, ultimately leading to fibrocartilage formation. While short-term symptom improvement can be achieved by treating OLT with a diameter less than 15 mm, it should be noted that fibrocartilage generated through MF exhibits limited mechanical elasticity and undergoes degradation over time [4–6]. In cases involving soft bone lesions with intact superficial cartilage, Retrograde Drilling presents distinct advantages as it allows for preservation of superficial cartilage integrity; however, careful control over drill bit positioning and depth is crucial to prevent any damage while achieving optimal depth [7, 8]. For OLT with large defects and associated subchondral bone cysts, both autologous osteochondral transplantation (AOT) and osteochondral allograft transplantation (OAT) can be considered as treatment options. The clinical efficacy of these two approaches is comparable; however, when compared to AOT, the use of OAT implants has been found to result in higher rates of cartilage wear and cyst formation on imaging [9]. Biological agents such as platelet-rich plasma and hyaluronic acid have emerged as novel treatments for OLT. Although there have been positive short-term outcomes reported in the literature, it is important to note that there is currently a lack of high-quality research supporting their long-term effectiveness [10, 11]. Additionally, there exist various chondrocyte implantation techniques such as autologous/allogeneic osteochondral transplantation, autologous chondrocyte implantation, and matrix-induced autologous chondrocyte implantation [12–14]. This technique involves a two-step process: Step 1 entails harvesting healthy cartilage tissue from non-weight-bearing areas and cultivating cartilage cells in vitro, while Step 2 involves implanting the in vitro-cultivated cartilage cells at the site of the cartilage defect. Although the outcomes are favorable, it is important to note that this technique necessitates two surgeries, leading to increased treatment costs and requiring a high level of surgical expertise. Consequently, it is not recommended as the primary choice for initial OLT treatment. A new technique for repairing cartilage defects by combining MF with collagen matrix scaffolds, called autologous matrix-induced chondrogenesis (AMIC), was introduced in 2003.AMIC allows the implantation of scaffolds with mesenchymalstem cells in a single operation, avoiding the need for laboratory culture of cells and a second implantation [15]. Behrens first introduced AMIC into OLT surgery in 2005 [16]. In the past few decades, some studies have reported that the AMIC technique has shown satisfactory results in treating primary OLT, secondary OLT (after initial surgical failure) and OLT with subchondral bone cysts [17–20]. Recently, several studies have integrated AMIC with autogenous bone grafting, particularly for the concurrent presence of subchondral cysts. These combined techniques aim to eradicate necrotic subchondral bone and employ bone grafting(BG) to uphold local vascular reconstruction and cartilage regeneration in the subchondral region of the talus [21–23]. Consequently, the fundamental surgical approaches for AMIC employed in this systematic review encompass not only MF but also BG. The previous systematic reviews on AMIC for the treatment of OLT have not specifically addressed mid-long term outcomes [17, 18]. The present study establishes a 2-year follow-up period as the designated threshold for intermediate follow-up.

This study aims to investigate the mid-long term efficacy of AMIC in treating OLT through a comprehensive literature review and meta-analysis. Our objective is to evaluate patient pain and functional outcomes, as well as surgical failure rate and complications. We hypothesize that AMIC treatment for OLT will continue to improve patient pain and function while demonstrating lower complication rates and surgical failures during mid-long term follow-up.

## Materials and methods

### Search strategy

The PubMed, Embase, and Web of Science databases were comprehensively searched up until November 11, 2023.The language used is exclusively English and there are no limitations on the publication date.Additionally, we scrutinized the reference lists of the included literature and early reviews to ensure that any studies overlooked during the electronic database searches were included. The following are the keywords utilized for conducting searches: AMIC or Autologous Matrix-Induced Chondrogenesis or collagen scaffold and osteochondral or cartilage and talus or talar.

### Eligibility criteria

The inclusion criteria for this meta-analysis were as follows: (1) patients diagnosed with osteochondral injury of the talus requiring autologous matrix-induced chondrogenesis treatment, (2) an average follow-up duration of 2 years or longer, and (3) one or more postoperative outcomes of interest such as visual analog scale (VAS), American Orthopaedic Foot & Ankle Society ankle-hindfoot score (AOFAS), Tegner, failure rate and complication. The exclusion criteria included: (1) incomplete data reports; (2) animal experiments, cell studies, reviews, meta-analyses, case reports, or conference abstracts.

### Data extraction and management

Two authors (Jiang and Li) screened a comprehensive screening of titles and abstracts, followed by a thorough examination of the full text based on predetermined inclusion criteria. Disagreement was resolved by consensus or a third author. They independently extracted relevant clinical information in a standardized format, encompassing: (1) author and publication year; (2) country; (3) study design; (4) patient characteristics such as age, gender, BMI, and lesion size; (5) sample size; (6) follow-up duration; (7) assessment measures including VAS score, AOFAS score, Tegner score, complications, surgical failure rate. The collected data were duplicated and systematically arranged in a Microsoft Excel spreadsheet.

### Assessment of methodological quality

Two senior authors (Jiang and Li) independently assessed the methodological quality of the selected studies.The risk of bias of cohort studies was assessed according to the Newcastle Ottawa Quality Assessment Scale (NOS) [24], which has a maximum score of 9 points attributed to the quality of selection (4 points), comparability(2 points), exposure(3 points), or outcome of study participants(3 points). Scores of 0–3, 4–6, and 7–9 were regarded as high, moderate, and low risk of bias, respectively. The risk of bias in the case series study was evaluated using the Joanna Briggs Institute (JBI) evaluation manual [25], which consists of 10 questions with response options including yes, no, unclear or not applicable.

### Statistical analysis

The extracted data were analyzed using Stata/MP 16.0(StataCorp). Continuous variables were presented as means and standard deviations. If partial continuous variables represented raw data, the calculations were converted to means and standard deviations for consistent result summarization. If mean or standard deviation values was not provided, they were derived from the median, minimum, and maximum values [26, 27]. The I^2^ statistic was employed to assess the heterogeneity among the included studies. Heterogeneity is considered insignificant when ranging from 0 to 40%, moderate between 30 and 60%, substantial between 50 and 90%, and considerable between 75 and 100%. Consequently, a fixed effect model is utilized when heterogeneity is below 60%; otherwise, a random effect model is employed. Significance tests were conducted with two-tailed criteria, considering *P* < 0.05 as statistically significant.

## Results

### Search results and characteristics of the included studies

**Fig. 1:**
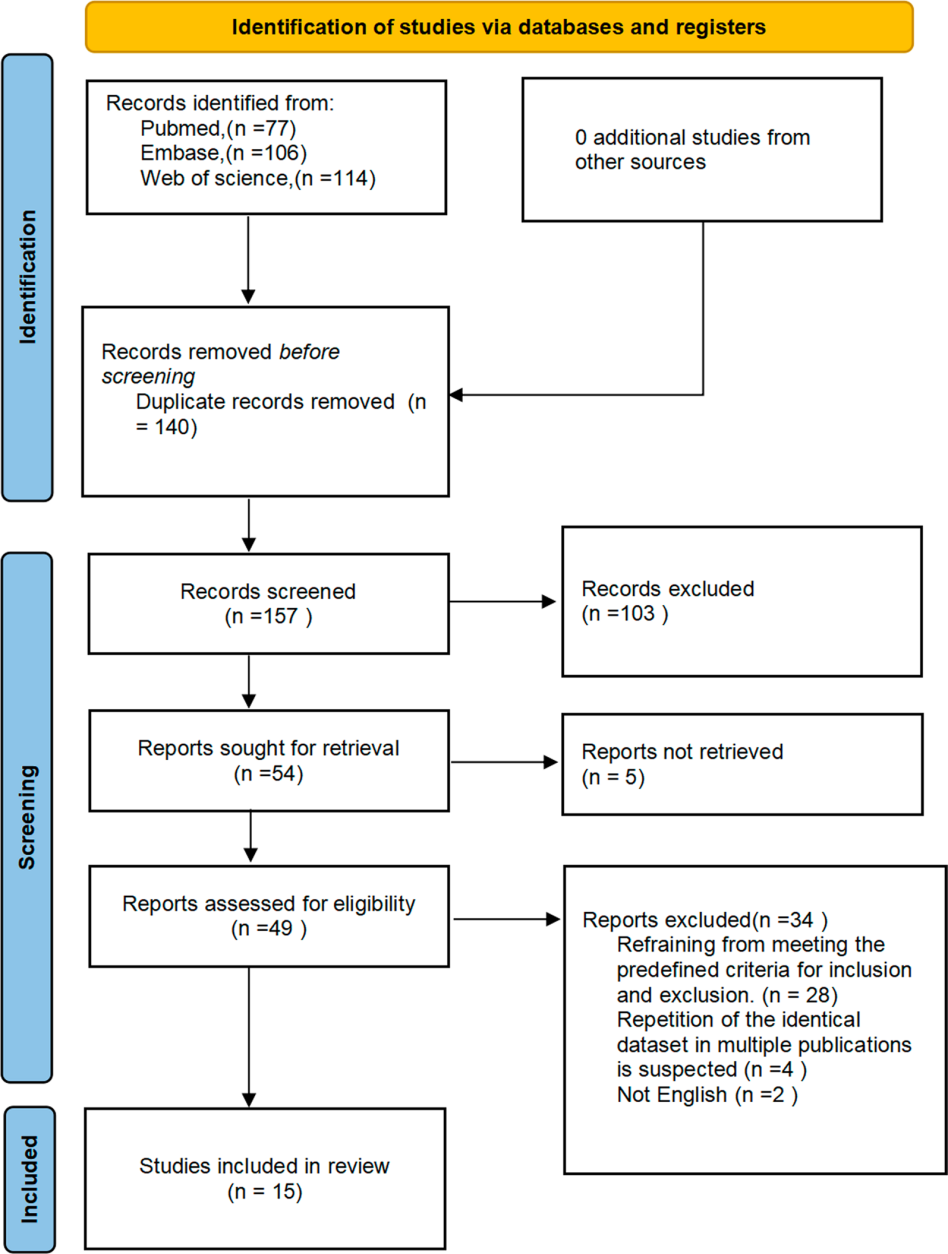
Flow diagram of the meta-analysis for the inclusion/exclusion of studies

A search of three major databases yielded a total of 297 studies (77 in Pubmed, 106 in Embase, and 144 in Web of Science). The exclusion process was conducted using Endnote20 software to remove 140 duplicate studies. Two investigators independently screened the remaining 157 articles by reviewing titles and abstracts, resulting in the exclusion of 103 irrelevant studies. Subsequently, the remaining 54 articles were assessed for full-text availability. Among them, five studies could not be obtained for full text, while another 28 studies were excluded based on the predefined inclusion and exclusion criteria. Additionally, four studies were identified as duplicates and two were not written in English. Finally, a total of fifteen eligible studies were included in the meta-analysis. The screening flow chart is in Fig. 1.

A total of 15 articles were included in this meta-analysis, comprising 12 case series involving 372 patients and 3 cohort studies involving 196 patients. These studies were categorized into the non-AMIC group (82 cases) and the AMIC group (114 cases). The characteristics of the included studies are available in Table 1.

**Table 1:** Basic characteristics of the included studies

	References	Country	ST	Simple size	Age (Year)	M/F	BMI (kg/m^2^)	Size (cm^2^)	FU (months)	Outcome
1	Yontar [28]	Turkey	CS	77	39.6(12–71)	40/37	27.2(19.4–40.9)	2.08 ± 0.66	35.5(6–92)	1,4
2	Wiewiorski [29]	Switzerland	CS	60	34.9 ± 11.5	24/36	27.6 ± 5.5	N/A	46.9 ± 17.8	1,3,4
3	Weigelt [30]	Switzerland	CS	33	35.1(13–75)	N/A	26.8 ± 4.3	0.9(0.4–2.3)	56.4(27.6–96)	1,3,4
4	Viehöfer [31]	Switzerland	CS	35	34.7 ± 15	19/14	28.7 ± 5.4	0.9 ± 0.6	54 ± 22.8	2,3
5	Valderrabano [32]	Switzerland	CS	26	33(17–55)	8/18	N/A	N/A	31(24–54)	1,2
6	Usuelli [33]	Italy	CS	20	36.1 ± 13.1	9/11	24.6 ± 2.7	1.3	24	1,2,4
7	Migliorini [34]	Germany	CT	AMIC = 52	31.5 ± 2.1	28/23	27.1 ± 6.4	2.8 ± 1.5	44.2 ± 19.9	1,2,4
				MF = 18	33.3 ± 6.2	10/8	26.9 ± 3.8	2.4 ± 0.4	41.5 ± 18.1	
8	Kubosch [35]	Germany	CS	17	38.8 ± 15.7	9/8	N/A	N/A	39.5 ± 18.4	1,2
9	Gorgun [36]	Turkey	CT	AMIC = 46	32.9 ± 7.6	24/22	N/A	N/A	69.3 ± 20.7	1,2
				BG = 48	31.7 ± 7.4	25/23				
10	D’Ambrosi [37]	Italy	CS	26	33.7 ± 11.0	17/9	24.5 ± 3.5	1.46 ± 0.72	42.6 ± 10.9	1,2
11	Becher [38]	Germany	CT	AMIC16	32.4 ± 12.5	7/9	22.6 ± 2.9	1.06 ± 0.47	68.4 ± 8.4	1,2
				MF = 16	33.3 ± 9.3	7/9	25.4 ± 4.9	1.11 ± 0.52	67.2 ± 6	
12	Ayyaswamy [39]	UK	CS	25	36(14–70)	14/11	N/A	N/A	24(14–70)	1,4
13	Albano [40]	Italy	CS	16	42.6 ± 18.4	8/8	26.3 ± 5.2	> 1.5	30 ± 16.9	1,2,4
14	Ackermann [41]	Switzerland	CS	13	33.4 ± 12.5	10/3	26.1 ± 3.7	0.8 ± 0.4	50.4 ± 19.2	3
15	Götze [42]	Germany	CS	24	46.75 ± 15.2	12/12	26.92 ± 5.7	N/A	25.17 ± 13.1	2

*ST* Study type; *FU* Follow-up; *CS* Case series; *CT* Cohort study; *AMIC* Autologous matrix-induced chondrogenesis; *MF* Microfracture; *BG* Bone grafting; *M/F* Male/female; 1 = VAS, 2 = AOFAS, 3 = Tegner, 4 = failure rate, N/A = Not available

### Study quality assessment

The JBI scoring methodology was employed in 12 case series, while the NOS scoring methodology was utilized in 3 cohort studies. All included studies demonstrated good methodological quality. The details of the included studies are listed in Tables 2 and 3

**Table 2:** JBI critical appraisal quality assessment of the case series study

References	Q1	Q2	Q3	Q4	Q5	Q6	Q7	Q8	Q9	Q10
Wiewiorski [29]	Y	Y	Y	Y	Y	Y	Y	Y	Y	Y
Viehöfer [31]	Y	Y	Y	Y	Y	Y	Y	N	Y	Y
Valderrabano [32]	Y	Y	Y	Y	Y	Y	N	Y	Y	Y
Kubosch [35]	Y	Y	Y	Y	Y	Y	N	Y	Y	Y
D’Ambrosi [37]	Y	Y	Y	Y	Y	Y	Y	Y	Y	Y
Ayyaswamy [39]	Y	Y	Y	Y	Y	Y	N	Y	Y	Y
Albano [40]	Y	Y	Y	Y	Y	Y	Y	Y	Y	Y
Yontar [28]	Y	Y	Y	Y	Y	Y	Y	Y	Y	Y
Usuelli [33]	Y	Y	Y	Y	Y	Y	Y	Y	Y	Y
Ackermann [41]	Y	Y	Y	Y	Y	Y	Y	N	Y	Y
Götze [42]	Y	Y	Y	Y	Y	Y	Y	Y	Y	Y

Q1. Were there clear criteria for inclusion in the case series? Q2. Was the condition measured in a standard, reliable way for all participants included in the case series? Q3. Were valid methods used for identification of the condition for all participants included in the case series? Q4. Did the case series have consecutive inclusion of participants? Q5. Did the case series have complete inclusion of participants? Q6. Was there clear reporting of the demographics of the participants in the study? Q7. Was there clear reporting of clinical information of the participants? Q8. Were the outcomes or follow-up results of cases clearly reported? Q9. Was there clear reporting of the presenting sites’s /clinics’s demographic information? Q10. Was statistical analysis appropriate? *Y*: Yes; *N*: No

**Table 3:** Quality assessment according to the Newcastle–Ottawa scale

References	Selection	Comparability	Exposure	Total score
Migliorini [34]	4	1	2	7
Gorgun [36]	3	1	2	6
Becher [38]	3	1	2	6

### Meta‑analysis results

#### VAS score

**Fig. 2:**
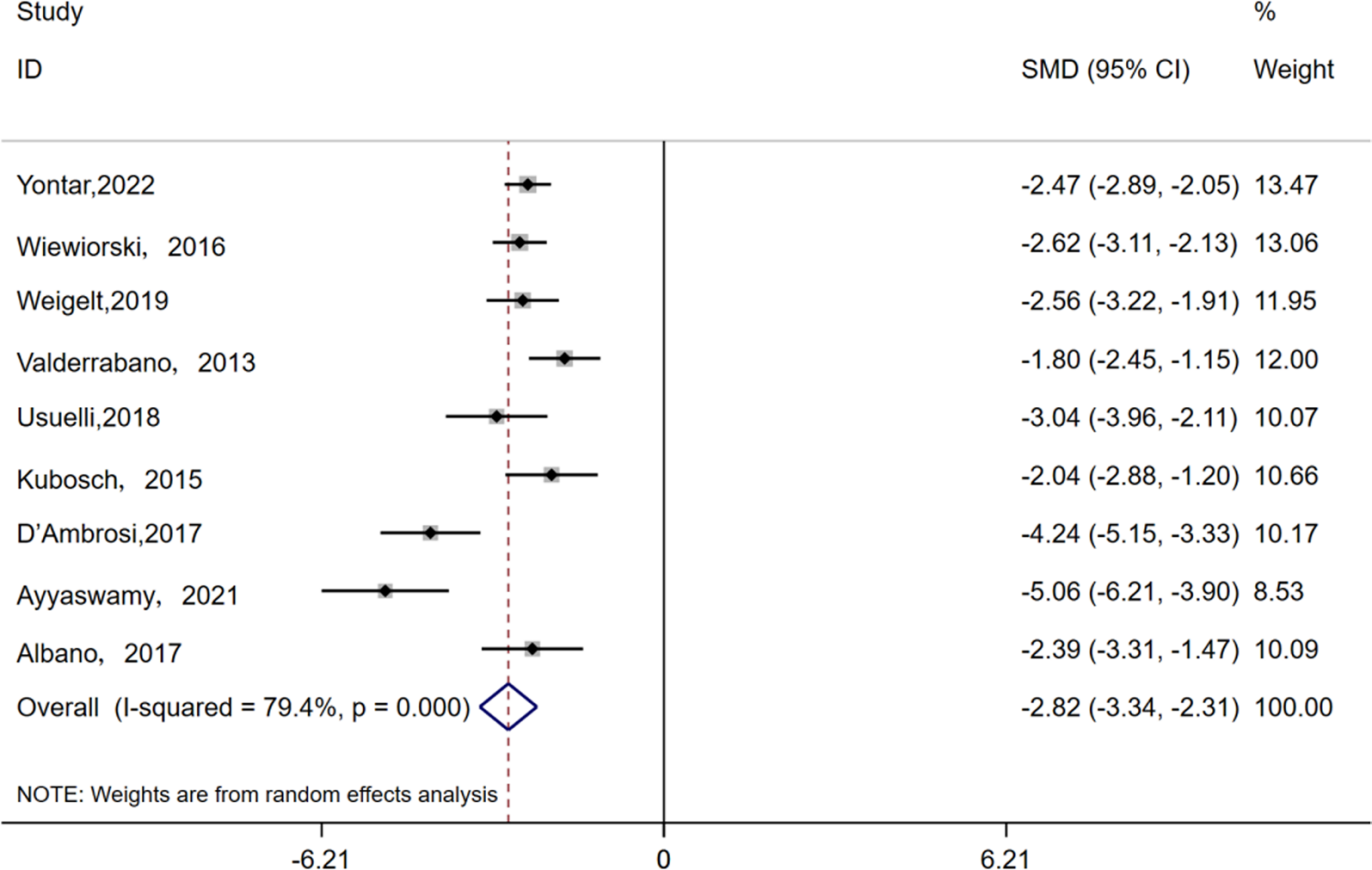
Forest plots for the change in VAS scores compared to baseline

The VAS scores were reported in 9 studies involving 305 patients, with an average follow-up duration of 37 months. Utilizing a random-effects model (I^2^ = 79.4%, *P* < 0.001), the analysis revealed a significant disparity in VAS scores between the final follow-up and preoperative assessments (SMD = − 2.825, 95% CI − 3.343 to − 2.306, *P* < 0.001), as displayed in Fig. 2.

#### AOFAS score

**Fig. 3:**
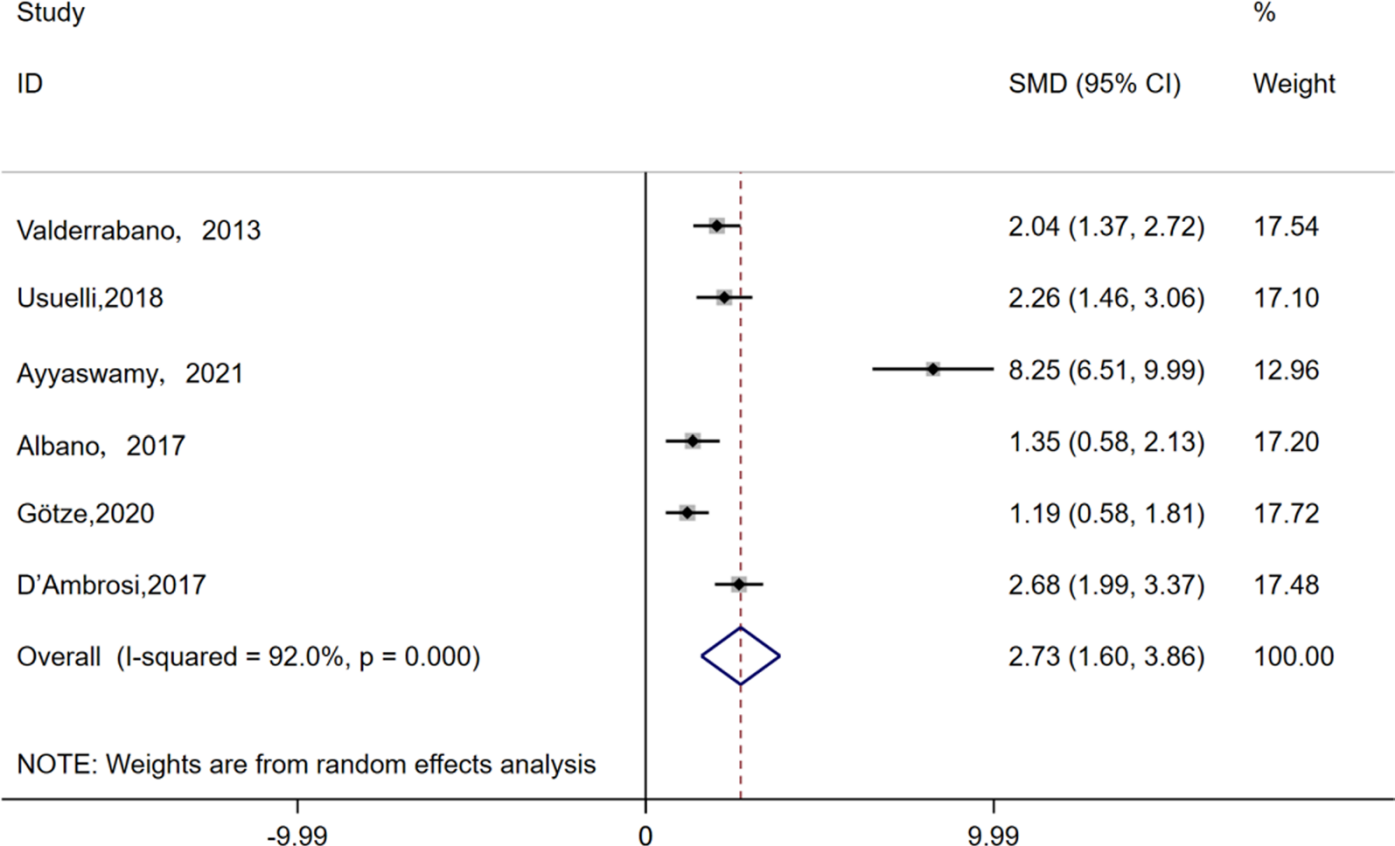
Forest plots for the change in AOFAS scores compared to baseline

The AOFAS score was reported in 5 studies involving a total of 142 patients, with an average follow-up duration of 29 months. Utilizing a random-effects model (I^2^ = 92.0%, *P* < 0.001), the analysis revealed a significant disparity in AOFAS scores between the final follow-up and preoperative scores (SMD = 2.73, 95% CI 1.60 to 3.86, *P* < 0.001), as shown in Fig. 3

#### Tegner score

**Fig. 4:**
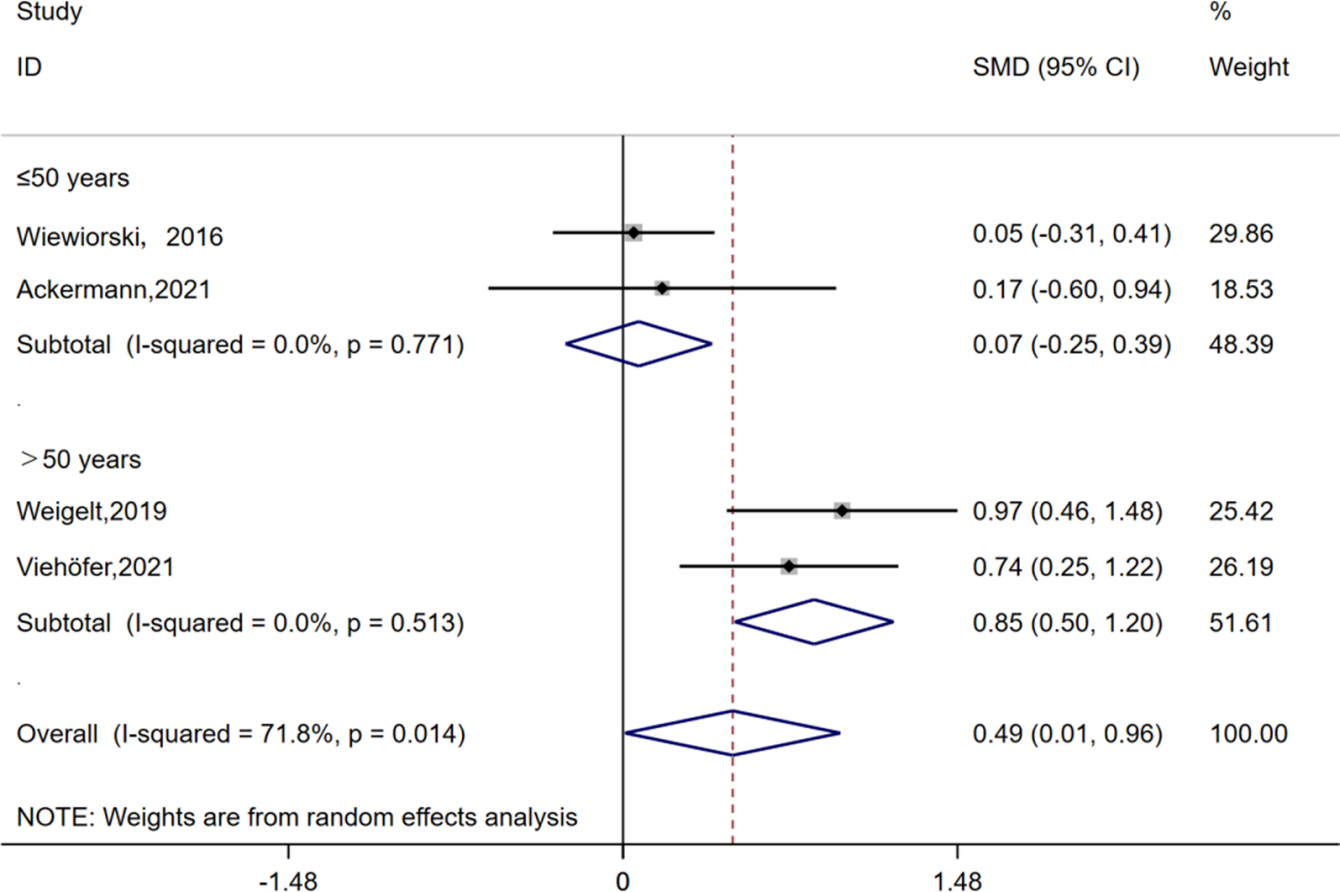
Forest plots for the change in Tegner scores and subgroup analysis

The Tegner score was reported in four studies involving a total of 141 patients, with an average follow-up time of 52 months. Utilizing the random effect model (I^2^ = 71.8%, *P* = 0.014), the analysis revealed a significant disparity in Tegner scores at the last follow-up compared to preoperative scores (SMD = 0.49, 95% CI 0.01 to 0.96, *P* = 0.046). Sensitivity results demonstrated that the combined outcomes of the meta-analysis remained robust and reliable even after excluding any individual study from consideration. Subsequent subgroup analysis based on age indicated no discrepancy in Tegner scores between preoperative and last follow-up among individuals under 50 years old (SMD = 0.07, 95% CI − 0.25 to 0.39, *P* = 0.673), with minimal heterogeneity within this group (I^2^ = 0%, *P* = 0.771). Conversely, there was a notable difference in Tegner scores between preoperative and last follow-up among individuals over 50 years old (SMD = 0.85, 95% CI 0.5 to 1, *P* < 0.001), also exhibiting low heterogeneity within this group (I^2^ = 0.0%, *P* = 0.513) (Fig. 4).

#### Failure rate

**Fig. 5:**
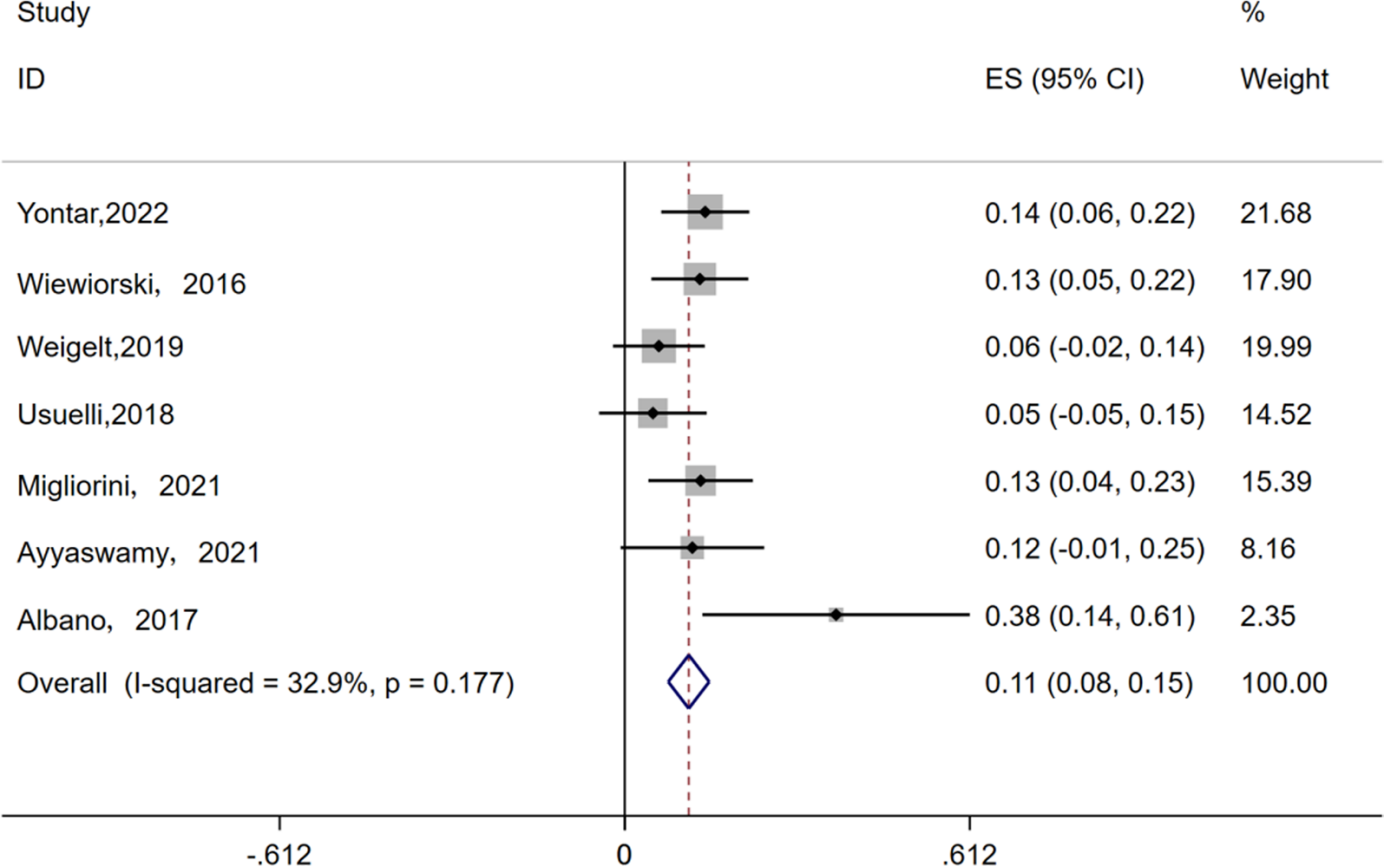
Forest plots for the failure rate

All 7 studies included in this meta-analysis, which included 283 patients, with an average follow-up time of 37 months, reported surgical failure. The level of heterogeneity between studies was low (I^2^ = 32.9%, *P* = 0.177); therefore, a fixed-effects model was used. As shown, the surgical failure rate was 11% (95% CI 8–15%) (Fig. 5).

#### AMIC vs non-AMIC

**Fig. 6:**
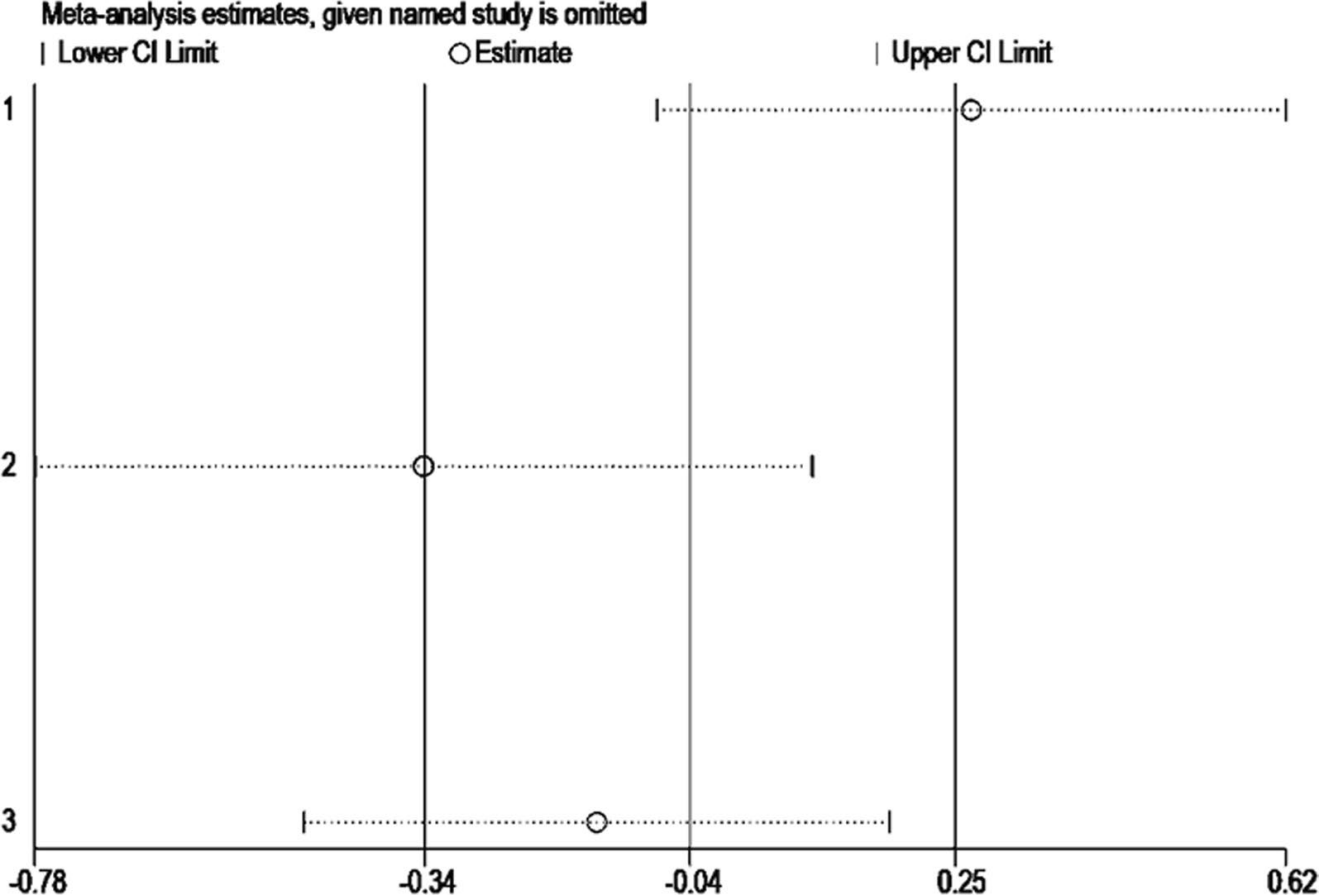
Results of sensitivity analysis

The VAS and AOFAS scores were compared between the AMIC group and non-AMIC group in 3 studies at the last follow-up. The AMIC group included 114 patients, with an average follow-up of 60 months, while the non-AMIC group had 82 patients with an average follow-up of 59 months. Significant heterogeneity was observed in VAS scores between the two groups (I^2^ = 81.8%, *P* = 0.004). Subsequent sensitivity analysis revealed that the meta-analysis results were influenced by 2 studies [34, 36]. Consequently, a decision was made to abandon the meta-analysis and only perform descriptive analysis (Fig. 6).

### Complications

Complications were reported in 5 studies involving 12 patients. 1 patient had delayed incision healing and no treatment was given; one patient had hypertrophic hyperplasia and underwent arthroscopic debridement;5 patients had persistent pain and underwent revision surgery, 2 patients with superficial portal skin infection received oral anti-inflammatory drugs, and 3 patients developed early arthritis and underwent arthrodesis, as shown in Table 4.

**Table 4:** The list of complications among the studies

Study	n	Complication	Procedure
Weigelt [30]	1	Delayed union	No procedure
Usuelli [33]	1	Hypertrophic proliferation	Arthroscopic removal and debridement
Migliorini [34]	5	Persistent pain for 4 years	Revision surgery
Gorgun [36]	2	Superficial skin infection of a portal	Oral antibiotic therapy
Ayyaswamy [39]	3	Early arthritis	Arthroscopic arthrodesis and their fusion

## Discussion

A total of 15 studies were included through literature retrieval and screening, with 12 case series being subjected to meta-analysis, while the remaining 3 cohort studies were descriptively analyzed. The findings demonstrated that AMIC treatment for OLT effectively alleviated pain symptoms, improved functional outcomes, and enhanced exercise capacity during medium to long-term follow-up. Furthermore, a less of complications were observed. However, this systematic review did not yield sufficient evidence to support the superiority of AMIC over simple MF.

The technique of MF involves the use of a specialized tool to create small holes in the subchondral bone at intervals of 3–4 mm. This allows for the release of bone marrow and blood, which then form blood clots to fill the defect site and promote fibrocartilage formation. However, it should be noted that these blood clots formed after MF lack sufficient mechanical resistance to remain in place [43]. In contrast, AMIC utilizes MF as part of a single-stage operation, followed by the application of a resorbable membrane over the treatment site. This membrane serves to protect and stabilize the blood clots containing factors stimulated by bone marrow release [44]. Although there are currently no comparative studies on the short-term efficacy of AMIC and MF, previous retrospective studies have demonstrated significant improvements in ankle function and pain symptoms in the short term for AMIC cases [33, 45, 46]. A recent systematic review [17] involving 323 patients also revealed significant improvements in patient prognosis between 1 and 2 years. Based on these aforementioned studies, it can be inferred that AMIC exhibits comparable short-term clinical efficacy to MF. Therefore, this systematic review primarily focused on evaluating long-term efficacy. On the one hand, we discussed the results of meta-analysis encompassing clinical scores, surgical failure rates, and complications; on the other hand, due to limited literature comparing AMIC and MF available at present, we extensively reviewed previous studies during our discussion to explore the clinical outcomes of both procedures.

Although MF can achieve favorable early outcomes for OLT [47, 48], its long-term efficacy remains unsatisfactory [5, 49, 50]. This may be attributed to the fact that the fibrocartilage formed after MF primarily consists of type I collagen, while chondrocytes in the ankle joint cavity are scarce and exhibit poor regenerative capacity. Consequently, achieving complete integration between fibrocartilage and primary cartilage becomes challenging, with fibrocartilage being structurally and biomechanically inferior to hyaline cartilage [4]. As a result, fibrocartilage undergoes degradation over time, as evidenced by increasing pain levels, revision rates, complication incidence, and extent of cartilage injury. As for the mid-long term efficacy of AMIC, our studies showed good clinical outcomes, with 3 studies with an average follow-up time of more than 4 years and 3 studies with an average follow-up time of more than 5 years, and all studies with a follow-up time ranging from 2 to 10 years. In addition, through the analysis of the existing literature, we found that the efficacy of AMIC would gradually increase over a period of time and remain stable for a long time after reaching a peak. Walther [17] posited that AMIC could significantly enhance the clinical outcomes of patients within a 5-year postoperative period. Efrimma [51], in 62 OLT patients with a median follow-up of 84 months, observed substantial improvements in VAS and AOFAS scores during the first 2 years after OLT surgery, while no significant changes were noted in clinical outcomes between the 24th and 60th month. This finding aligns with Gottschalk's study [52], which reported significant enhancements in pain relief and functional recovery following AMIC, particularly within the initial year and peaking at two years. Moreover, these improvements were sustained for at least five years, accompanied by notable restoration of mobility and further enhancement of patient satisfaction at the five-year follow-up point. Collectively, The findings of these studies demonstrate that AMIC exhibit a consistently positive long-term outcome. However, due to the low quality of these studies, further high-quality research is needed to validate them.

As for postoperative complications and surgical failure rate, Walther's systematic review [17] included 4 studies with a total of 6 patients who underwent revision surgery due to persistent pain caused by articular fibrosis, hypertrophic scar tissue, or progression of degenerative arthritis. Migliorini's systematic review [18] reported that 7.8% of patients required revision surgery. In our meta-analysis of 7 studies, we observed similar complications as in other systematic reviews. However, when it comes to the surgical failure rate, our meta-analysis yielded a final result of 11%, which significantly differs from previously published systematic reviews. Through analysis, on one hand, the definition of surgical failure in this study is relatively broad. For studies that have not clearly reported the failure rate, we also include the dissatisfaction rate in the category of failure rate. On the other hand, our study did not provide a clear definition for OLT's nature. For instance, Yontar's study included primary OLT [28], primary with tumor-related OLT, and revision OLT. The surgical failure rates were 4.8%, 11.8%, and 38% respectively for these categories. Additionally, Albano's study reported a surgical failure rate as high as 38% [40]. It should be noted that MaioRegen® scaffold was used in this particular study (a cell-free biomimetic scaffold composed of type I collagen and hydroxyapatite), while most published studies on AMIC utilized Chondro-Gide® scaffold (a bilayer collagen I/III matrix scaffold). Another author observed through MRI and CT scans that incomplete cartilage repair and poor subchondral bone repair were induced by the MaioRegen® scaffold [53], which contributed to its high failure rate. In conclusion, we think that the choice of scaffold material can impact clinical outcomes; therefore we recommend using Chondro-Gide® scaffold based on the above research.

In terms of the efficacy of AMIC and MF, Migliorini et al.[34]reported that after an average follow-up period of 43.5 months for OLT defects measuring 27.1 ± 6.4 cm^2^, the clinical scores (AOFAS, VAS, Tegner) in the AMIC group were significantly superior to those in the MF group, with a notably lower failure rate compared to the latter group. Becher et al. [38], on the other hand, investigated OLT defects measuring 1.06 ± 0.47cm^2^ and found that although the average score in the AMIC group was better than that in the bone marrow stimulation(BMS) alone group at a five-year follow-up, this difference did not reach statistical significance. Upon analysis, it appears that one potential factor contributing to these divergent findings could be lesion size; thus prompting us to inquire whether lesion size influences the efficacy of AMIC. The study conducted by Chuckpaiwong et al. [54] examined 105 OLT patients who underwent MF treatment and observed that all patients with treatment failure had lesions larger than 15 mm, leading to the conclusion that membrane scaffolds were necessary for OLT > 1.5 cm^2^ [55]. However, a systematic review indicated that BMS as the sole treatment should be limited to osteochondral lesions smaller than 1 cm^2^ [56]. For surgical treatment of OLT > 1 cm^2^, the International Consensus Group on Cartilage Repair agreed that implanting scaffolds would yield superior and more reliable outcomes [57]. In 2024, the latest guidelines issued by the German Society of Orthopedics and Traumatology (DGOU) stated that the long-term benefits of scaffolds were proportional to the size of the lesion; in other words, larger areas exhibited more significant effects [58]. Therefore, based on existing research [23, 27], DGOU recommended adding additional scaffolds when OLT > 1 cm^2^ (instead of 1.5 cm^2^).

Another point of interest for us was age. Ayyaswamy [39] observed in his study that there was no significant correlation between age and AOFAS score, and the correlation between age and VAS score was only weak to moderate, but not statistically significant. Therefore, he concluded that age did not have a significant impact on outcome scores. However, another scholar presented a different perspective. Efrima [51] discovered significant differences in the Short Form-12, Halasi, and the University of California at Los Angeles scores between patients younger than 33 years old and those older than 33 years old at the 60-month follow-up period, suggesting that increasing age was significantly associated with poorer outcomes. D'Ambrosii [59] also found in his study that younger patients had significantly better functional recovery compared to older patients. Interestingly though, we subgrouped the Tegner scores of 141 patients from four studies based on their ages (with 50 years old as the threshold). At the final follow-up assessment, we observed that the exercise level of patients younger than 50 years old remained unchanged compared to pre-surgery levels; however, the exercise level of patients older than 50 years old showed a positive improvement after undergoing AMIC treatment, indicating its effectiveness in restoring exercise capacity among older individuals.

Despite these significant findings, the study has certain limitations. Firstly, the sample size of the studies included in this analysis was small and there was a lack of control groups. Therefore, our evaluation focused on efficacy and risk, without clear evidence demonstrating AMIC's superiority over simple MF. Secondly, the majority of our included studies are case series, which would indicate a high risk for bias and these studies were conducted in European countries such as Switzerland and Germany, which resulted in homogeneity in geographical origin and medical institutions. Hence, caution is required when extrapolating these results to a broader population.

## Conclusion

The present meta-analysis revealed that the choice of stent utilized in the AMIC procedure, along with patient age and OLT area size, exerted an impact on the ultimate clinical outcome. To a certain extent, it substantiated a good mid-long term therapeutic efficacy of AMIC in ameliorating pain, function, and exercise levels among OLT patients. Nevertheless, future endeavors should focus on conducting further high-quality research to more comprehensively evaluate its effectiveness in treating OLT.

## Data Availability

Data and materials will be available on reasonable request.
